# A Pedunculated Cervical Mass: A Case Report

**DOI:** 10.22038/IJORL.2022.59029.3037

**Published:** 2022-03

**Authors:** Mohammad Reza Afzalzadeh, Amir Bahador Sadri, Masoumeh Hosseinpoor, Mohammad Karimpour Malekshah

**Affiliations:** 1 *Sinus and Surgical Endoscopic Research Center, Mashhad University of Medical Sciences, Mashhad, Iran.*; 2 *Department of Otorhinolaryngology, Head and Neck surgery, Mashhad University of Medical Sciences, Mashhad, Iran.*

**Keywords:** Accessory tragus, Branchial arch, Congenital cartilaginous rest of the neck, Sternocleidomastoid

## Abstract

**Introduction:**

A congenital cervical mass is a considerable health problem worldwide; however, accessory tragus (AT) in the neck is extremely rare. The cervical variant of AT or congenital cartilaginous rest of the neck (CCRN) is a rare anomaly related to the branchial arch located at the lateral of the neck that typically presents as an asymptomatic papule or nodule along the anterior border of sternocleidomastoid (SCM) muscle. It is detected since birth or in the first few years of life. Diagnosis is based on the clinical characteristics of the lesion, surgical findings, and histopathologic studies.

**Case Report:**

A young man with no underlying diseases or known congenital anomaly was referred by a dermatologist for an asymptomatic pedunculated papule in the left mid-cervical area. Physical examination reveals a firm and mobile papule with a size of 1*1 cm on the anterior middle 1/3 border of the SCM. Radiologic findings illustrated a mass nearby the SCM with a long tract beneath it extending upward. The lesion was finally resected, and during surgery, a long tract was discovered, and histopathologic examination confirmed the diagnosis of a CCRN.

**Conclusions:**

***Although rare, the cervical variant of AT or CCRN should be considered in a differential diagnosis of benign masses in the neck.***

## Introduction

Accessory tragus (AT) is the most prevalent external ear anomaly that was first described by Birkett in 1858 (1). It usually occurs in the preauricular region. The cervical area is an uncommon site for AT (1). The cervical variant of AT is called congenital cartilaginous rest of the neck (CCRN). It is also known as wattle, cervical auricle, branchial fibrochondroma, congenital branchial arrest, cervical tab, Meckel's cartilaginous remnant, and elastic cartilage choristoma of the neck (2,3). In this study, we present a young man with an asymptomatic pedunculated cervical lesion that was referred by a dermatologist to the ENT clinic for surgical removal. He was finally diagnosed histopathologically with CCRN.

## Case Report

**Fig 1:**
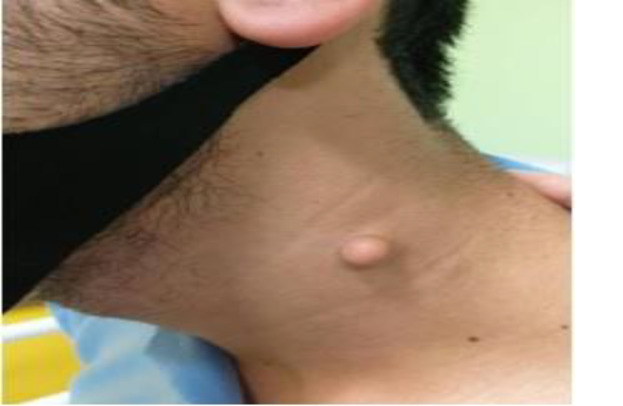
Macroscopic findings showing a pedunculated and firm papule located at the left lateral of the neck

A 21-year-old male presented with a chief complaint of a left neck mass since childbirth. His history included an uneventful full-term pregnancy, normal vaginal delivery, and no other congenital defects. Family and medical histories had no remarkable findings. There were no other congenital deformities and no complaint of hearing loss. Routine head and neck region examination revealed a mass at the level of thyroid cartilage on the anterior middle 1/3 border of sternocleidomastoid (SCM) (Figure 1). The mass had changed neither in size nor in consistency since childhood. The patient had not reported a history of infection or inflammatory changes in his neck mass. It was painless with no fluctuation in size or discharge. It was 1*1 cm in dimension with smooth surface texture and normal surrounding skin. Moreover, it was not moving with tongue protrusion.

In palpation, it was mobile, firm in consistency with no tenderness or erythema, and also no discharge in compression. No regional lymphadenopathy was palpated. Spiral neck CT scan illustrated a 9*4-mm soft tissue density containing a sinus tract which was located on the left middle 1/3 of the neck extended through SCM at the level of the thyroid cartilage to the hyoid bone (Figure 2). 

**YAour text hereFig 2. A:**
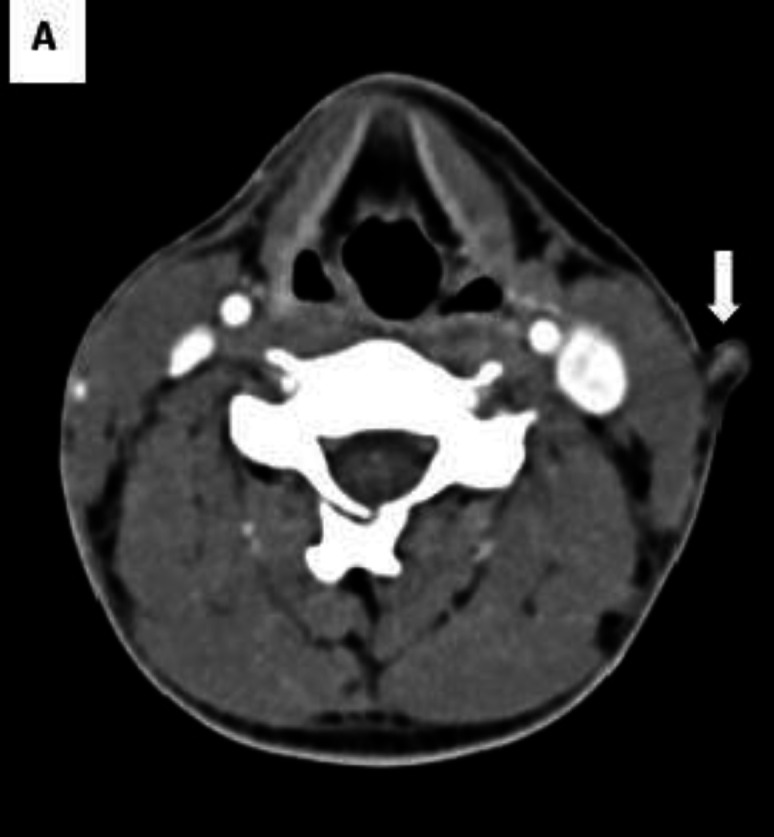
Spiral neck CT scan, axial view. The lesion with central cartilaginous density has been shown with an arrow

**Fig 2. B:**
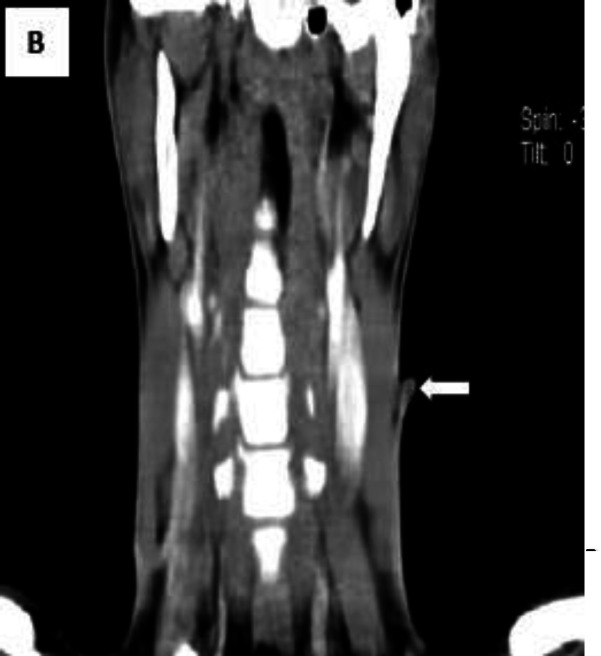
Spiral neck CT scan, coronal view. The sinus tract has been shown with an arrow as a low-density area just beneath the lesion

**Fig 2 C:**
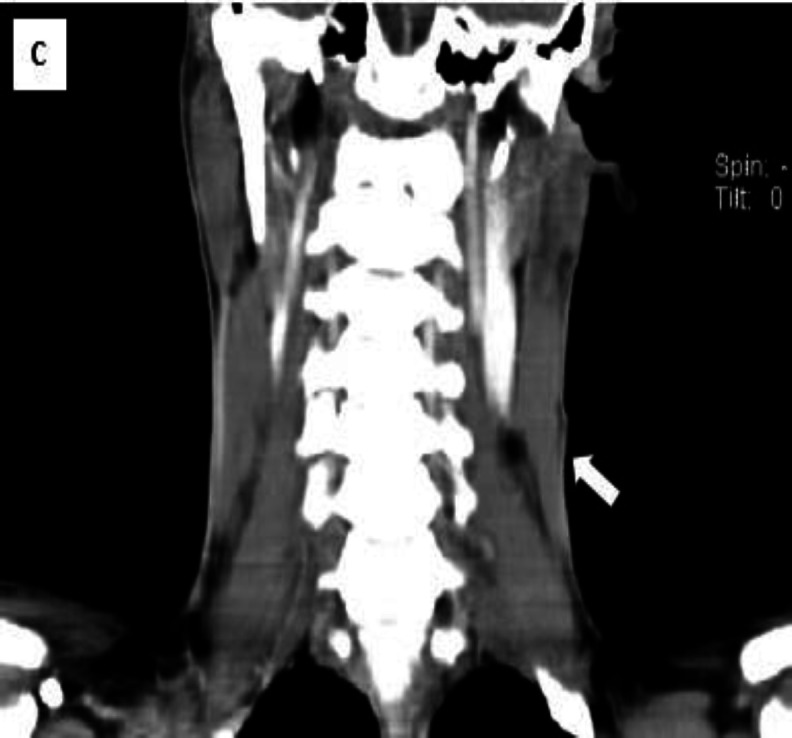
Spiral neck CT scan, coronal view. The tract extended superficially through SCM (the arrow)

**Fig 3:**
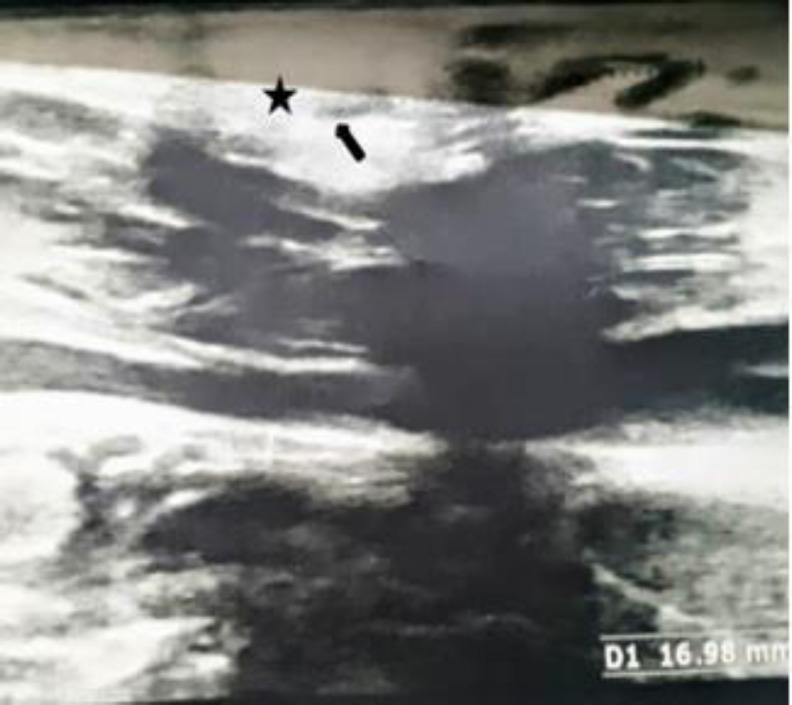
Sonographic findings showing the lesion (the asterisk) and sinus tract (the arrow)

The sonographic findings illustrated a mixed echo 4*8.5-mm lesion with small cystic particles, definite margins without vascular components superficial to SCM, with a 17-mm long tract beneath it. This tract arch was about 6 mm superiorly but did not penetrate underneath the muscle (Figure 3).

**Fig 4:**
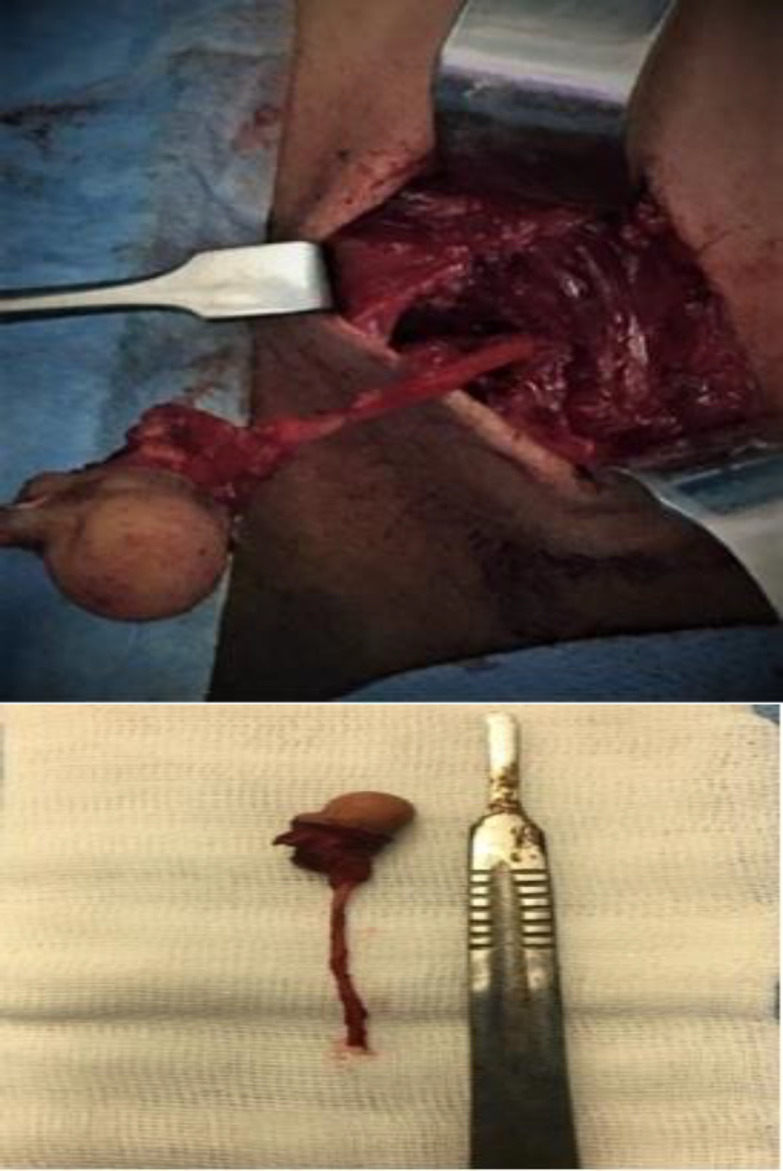
Left: Intraoperative findings illustrating a tract just beneath the lesion extending upward along the anterior border of the sternocleidomastoid muscle. The mass was excised via elliptical incision, the tract followed and ligated nearby the anterior belly of the digastric muscle. Right: Surgical excision of the lesion and tract

It was completely resected by an adequate excision via an elliptical incision. There were cartilaginous fragments in the center of the lesion. Furthermore, there was a tract laid beneath the lesion which was extending upward. The tract was exposed as much as possible, and it terminated at the level of the hyoid bone next to the anterior belly of the left digastric muscle (Figure 4). 

**Fig 5:**
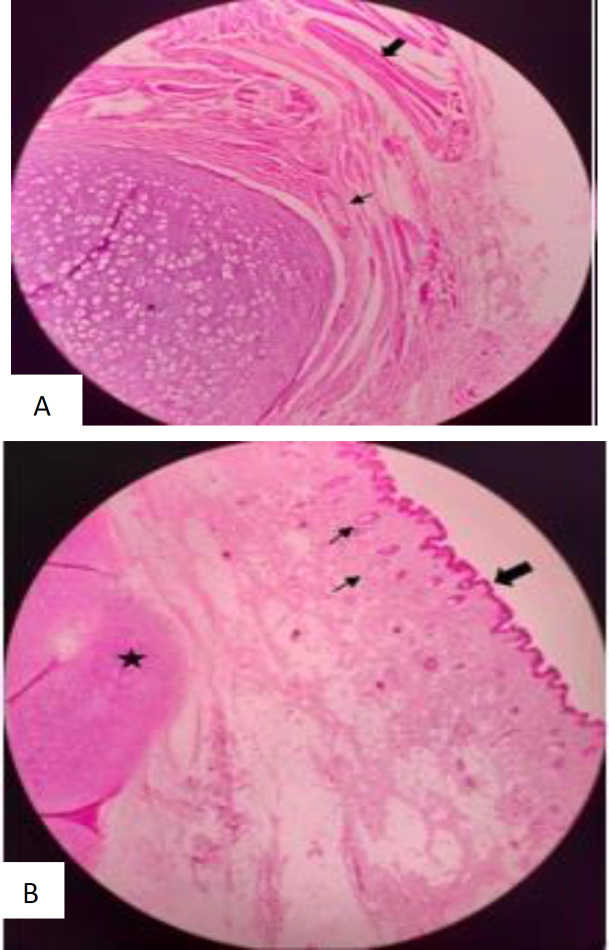
A: Epidermis (the large arrow), the dermis contains pilosebaceous follicles, sweat glands (the small arrow), and hyaline cartilage beneath (the asterisk). Left: Straight muscle (the large arrow) and neuron bundles (the small arrows) around cartilage tissue (the asterisk)

The microscopic findings demonstrated a polypoid lesion covered by epidermal tissue with pilosebaceous follicles, sweat glands, and adipose tissue in the dermis, as well as a hyaline cartilage tissue just beneath the dermis. Straight muscle and neuron bundles were also observed in the depth of the lesion around the cartilage tissue (Figure 5). These findings were compatible with CCRN.

## Discussion

CCRN is a rare manifestation of AT and branchial arch maldevelopment (1,3). Moreover, it is a subcutaneous firm papule or nodule that can be sessile or pedunculated (3, 2). It is regularly unilateral; however, bilateral cases have rarely been reported (4). Overall, CCRN does not change in size but may grow up to 20 cm in diameter which is more common in males (M/F: 2:1) (2); nevertheless, literature described the sex distribution equally (3). 

In addition, it is detected at birth or in the first few years of life but can be neglected for a long time as occurred in our case (5,6). It is generally an isolated finding; however, it can be accompanied by various syndromes (e.g., Goldenhar’s syndrome, Treacher Collins, Townes- Brocks, Wolf-Hirschhorn, and Delleman syndromes), first and second branchial anomalies, malformation of the ear (e.g., microtia and external ear canal stenosis), as well as cardiovascular, nervous, gastrointestinal, genitourinary, musculoskeletal, respiratory, visual, and endocrine complications (7,8). Consequently, audiometry and screening for these syndromes and anomalies are necessary (1). The incidence of accompanying syndromes and anomalies varies based on different studies. Nonetheless, Woo et al. confirmed that approximately one-third of patients with CCRNs have associated congenital anomalies (7). CCRN embryogenesis is debated, and it originates from a branchial remnant or the auricular cartilage. Both theories agree that it is the derivation of the branchial arch (5). Embryologically, in four weeks of gestation, the auricle rises to develop from the first (mandibular) and second (hyoid) branchial arches. During the fifth and sixth weeks, the first and second arches form six mesenchymal tubercles called the hillocks of His. Three hillocks appear on each arch, and as they develop, they ultimately fuse to form the auricle. As the mandible grows, the primitive auricle ascends from the lower lateral neck to the original site in the head. Therefore, AT is found in the migratory route of the first two branchial arches mostly near the tragus (periauricular region) and seldom on the lateral neck along the anterior of the SCM (4). 

Mukai et al. showed that preauricular and cheek AT was overall derived from the first branchial arch; otherwise, cervical AT or CCRN was a derivation of the second branchial arch. CCRN is usually presented in the lower one-third of SCM; however, in our case, it is presented in the middle one-third (9). Clinical features and surgical findings are helpful in the diagnosis of CCRN, but a definite diagnosis is based on histopathologic studies (3). 

The histological examination shows elastic cartilage surrounded by the eccrine gland, adipose tissue, pilosebaceous units, and hair follicles (5). The differential diagnoses of CCRN include thyroglossal duct cyst, thymic cyst, branchial cleft, hair follicle nevus, congenital midline hamartoma, fibroepithelial polyp, epidermoid cyst, and squamous papilloma (5). The presence of cartilage is a characteristic finding; however, it is not always present (it is observed in 78.4% of cases). It helps to distinguish it from other differential diagnoses (5,9). 

CCRN is a benign and symptomless lesion, the chance of infection is low; therefore, no treatment is needed, reassurance is sufficient, and surgery is done just for cosmetic reasons, or if it is irritated (1,8), it preferably should be done before school ages for social purposes (7). The most important principle in surgery is complete excision of cartilage component; otherwise, it can cause recurrence, surgical wound infection, interference in the healing process, and post-surgical chondritis/ chondrodermatitis (1,3,5). Consequently, the shave excision is forbidden due to the risk of cartilage remanent and complications (2). In some cases, as noted in our case, CCRN has a fibrotic band or tract that communicate the lesion to underlying fascia or it may communicate with branchial cyst or sinus, platysma, or SCM muscle; therefore, imaging, such as ultrasonography, is recommended if surgery is indicated to assess the presence of tract, surgery extension, and exclusion of cyst or fistula (5,8). The recurrence after surgery is low, and in multiple reports, no recurrences were observed (10).

## Conclusion

Although incidence in the neck is rare, AT should be considered.
